# ETH1/395: From the "Digital Disease" on to the Hippocratic Nosos: Helping the physician to reinstate the individual patient in compartmentalized medicine

**DOI:** 10.2196/jmir.1.suppl1.e41

**Published:** 1999-09-19

**Authors:** B Spyropoulos, G Papagounos

**Affiliations:** ^1^Technological Educational Institution of AthensAthensGreece

**Keywords:** Medical Ethics, Decision Support, Medical Education

## Abstract

**Introduction:**

Medical data, disseminated in the Web or available in other digital forms, usually include clinical information obtained by the case history, signals related to bodily functions which are collected from in vivo diagnostic procedures, data acquired through in vitro diagnostic tests, images related to the morphology and the functions of the body and, finally, information related to various therapeutic interventions. Although these data, on the one hand, constitute cost-effective and practical means, augmenting equality in medical training, on the other, they result in a new type of fragmentation and compartmentalization of the patient's body and personality, thus endangering the interpersonal relation between him and the physician. Further, the use of the Web, combined with other traditional ways of distribution of medical data, such as published epidemiological studies and case-studies presentation in conferences and textbooks, intensifies the risk of eliminating the individual characteristics of the specific patient, in favor of the impersonal condition.

**Methods:**

The main challenge of a decision supporting system employing various educational means in web-sites or elsewhere, is the incorporation of social and ethical premises into the mode of the presentation of the medical inferences employed. This, however, necessitates the identification of the ethical issues which are involved in decisions made in a health care context and it requires the codification and the classification of the ethical problems. Additionally, this procedure should be employed in all sectors and the ensuing activities of the health-care providers, since problems are not identical nor do they carry the same weight in all real-world incidents.

**Results:**

The authors argue, on the basis of a ten-year period of experience in the inter-disciplinary education of health-care professionals, that this goal can be reached only by the appropriate systematic instruction of all those -in the contemporary health-care system- involved in decision-making training. This means that the methods of reasoning employed in the inferences made in the various medical specialties should become common knowledge and that the acquaintance with the social and ethical issues, present in health care delivery, should be sine qua non.

**Discussion:**

Such an educational process would contribute to the promotion of inter- disciplinary research and it would address, in an effective manner, the problem of intra-specialty communication in medicine. It would permit also the demonstration of the common characteristics of the reasoning models employed since the time Hippocrates.

